# Genome wide identification and characterization of fertility associated novel CircRNAs as ceRNA reveal their regulatory roles in sheep fecundity

**DOI:** 10.1186/s13048-023-01178-2

**Published:** 2023-06-20

**Authors:** Salsabeel Yousuf, Waqar Afzal Malik, Hui Feng, Tianyi Liu, Lingli Xie, Xiangyang Miao

**Affiliations:** 1 Institute of Animal Sciences, Chinese Academy of Agricultural Sciences, Beijing, 100193 China; 2 Agricultural Genomics Institute at Shenzhen, Chinese Academy of Agricultural Sciences, Shenzhen, 518120 China

**Keywords:** Non-coding RNAs, Competitive endogenous RNA (ceRNA), Reproduction, Genomic organization, Expression profiling

## Abstract

Reproductive traits play a vital role in determining the production efficiency of sheep. Maximizing the production is of paramount importance for breeders worldwide due to the growing population. Circular RNAs (circRNAs) act as miRNA sponges by absorbing miRNA activity through miRNA response elements (MREs) and participate in ceRNA regulatory networks (ceRNETs) to regulate mRNA expression. Despite of extensive research on role of circRNAs as miRNA sponges in various species, their specific regulatory roles and mechanism in sheep ovarian tissue are still not well understood. In this study, we performed whole genome sequencing of circRNAs, miRNA and mRNA employing bioinformatic techniques on ovine tissues of two contrasting sheep breeds "Small tail Han (X_LC) and Dolang sheep (D_LC)", which results into identification of 9,878 circRNAs with a total length of 23,522,667 nt and an average length of 2,381.32 nt. Among them, 44 differentially expressed circRNAs (DECs) were identified. Moreover, correlation between miRNA-mRNA and lncRNA-miRNA provided us with to prediction of miRNA binding sites on nine differentially expressed circRNAs and 165 differentially expressed mRNAs using miRanda. miRNA-mRNA and lncRNA-miRNA pairs with negative correlation were selected to determine the ceRNA score along with positively correlated pairs from lncRNA and mRNA network. Integration of ceRNA score and positively correlated pairs exhibit a significant ternary relationship among circRNAs-miRNA-mRNA demonestrated by ceRNA, comprising of 50 regulatory pairs sharring common nodes and predicted potential differentially expressed circRNAs-miRNAs-mRNAs regulatory axis. Based on functional enrichment analysis shortlisted key ceRNA regulatory pairs associated with reproduction including circRNA_3257-novel579_mature-EPHA3, circRNA_8396-novel130_mature-LOC101102473, circRNA_4140- novel34_mature > novel661_mature-KCNK9, and circRNA_8312-novel339_mature-LOC101110545. Furthermore, expression profiling, functional enrichments and qRT-PCR analysis of key target genes infer their implication in reproduction and metabolism. ceRNA target mRNAs evolutionary trajectories, expression profiling, functional enrichments, subcellular localizations following genomic organizations will provide new insights underlying molecular mechanisms of reproduction, and establish a solid foundation for future research.

**Graphical Abstract:**

Graphical abstract summarizing the scheme of study

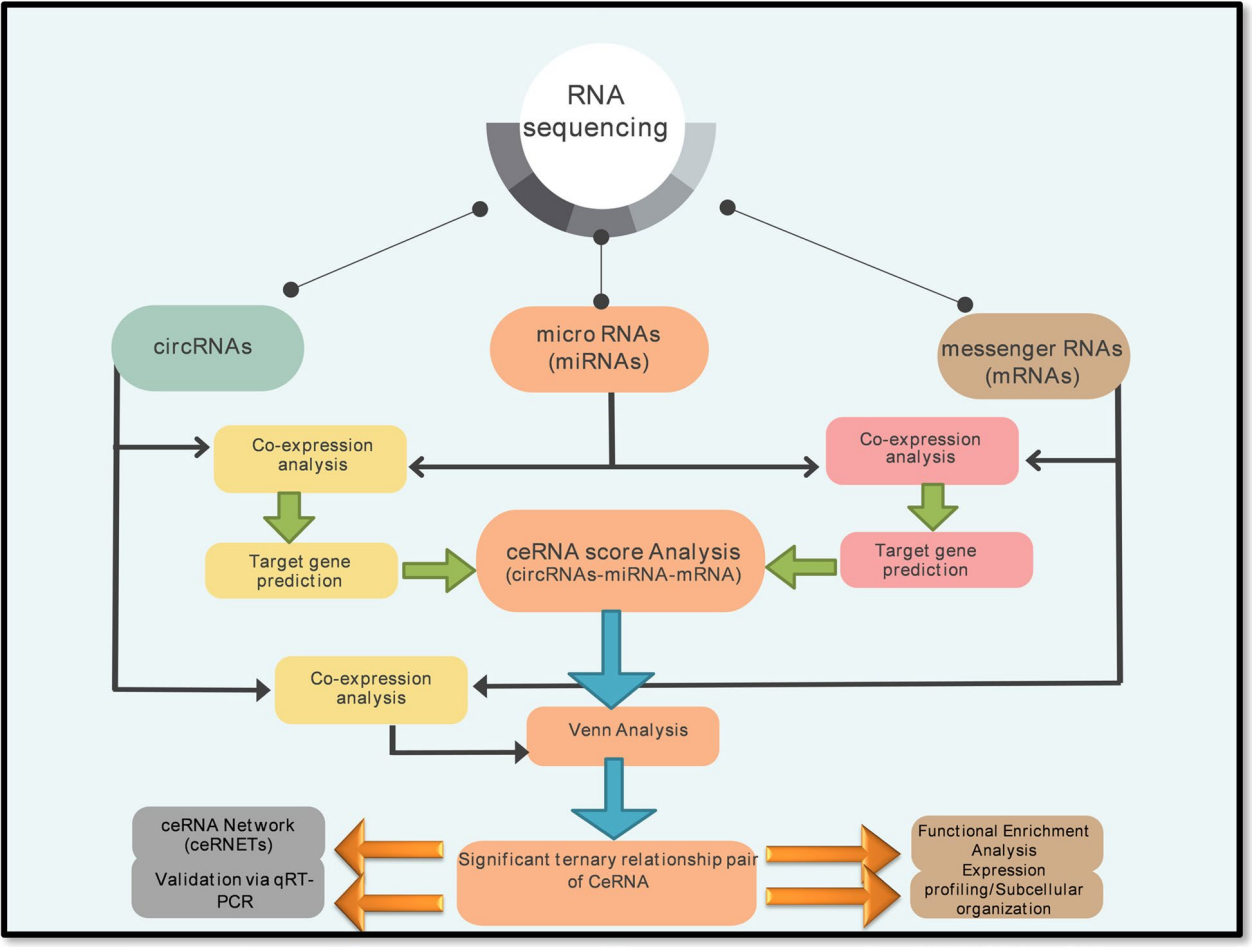

**Supplementary Information:**

The online version contains supplementary material available at 10.1186/s13048-023-01178-2.

## Introduction

Reproductive traits, such as prolificacy, multiple births, and fecundity, are threshold and crucial for the profitability of sheep production but have low heritability rate [1–3]. Therefore, developing reproductive traits has become a significant interest in sheep breeding [4], as it can increase the production of sheep-derived products, ranging from wool to lamb meat [5]. The genetic background of a ewe primarily determines its fertility, which depends on various factors, including ovulation rate, estrus cycle, and litter size, with the fecundity of the ewe playing a central role, mainly concentrated on the ovary [5–7]. Of note, several studies have demonstrated that follicular development and ovulation can be controlled by the set of single or multiple genes with major and minor effects [8, 9]. BMPR1, BMP15, GDF9, and B4GALNT2 were well known for their contribution to high prolificacy [3, 10, 11]. However, lack of knowledge of the genetics behind the complicated physiological mechanisms governing fertility hinders selection accuracy.

Reproductive traits, such as prolificacy, multiple births, and fecundity, are crucial for sheep production profitability but have a low heritability rate [1–3]. Therefore, developing reproductive traits has become a significant interest in sheep breeding [4], as it can increase the production of sheep-derived products, ranging from wool to lamb meat [5]. The genetic background of a ewe primarily determines its fertility, which depends on various factors, including ovulation rate, estrus cycle, and litter size, with the fecundity of the ewe playing a central role, mainly concentrated on the ovary [5, 7]. Several studies have shown that follicular development and ovulation can be controlled by the set of single or multiple genes with major and minor effects, including BMPR1, BMP15, GDF9, and B4GALNT2, which are known for their contribution to high prolificacy [3, 10, 11]. However, the lack of knowledge regarding the genetics behind the complicated physiological mechanisms governing fertility hinders selection accuracy.

Non-coding RNAs (ncRNAs), including microRNAs (miRNAs), long non-coding RNAs (lncRNAs), and circular RNAs (circRNAs), widely participate in post-transcriptional gene expression regulation and several biological processes [12–14]. Circular RNAs (circRNAs) are closed long non-coding RNAs that are formed by the back-splicing of exons from a single pre-mRNA, in which 5' and 3' termini are covalently bonded [15]. CircRNAs are extensively conserved, widely expressed in mammalian cells and often exhibit cell type- or tissue-specific expression patterns [16, 17]. Increasing evidences suggested that circRNAs have been implicated in the regulation of a number of biological processes and participate in various pathophysiology processes [18]. The biological role of circRNA is serving as a decoy for microRNA or competing ceRNA [19–21]. This is the most widely studied function of circRNAs that sponge and inhibit relevant miRNA by complementary base paring. They can release the inhibitory effect of miRNAs on their targeted mRNAs, thereby promoting the transcription and protein translation of target genes. Salmena et al. introduced a systematic "ceRNA-hypothesis", in which circRNAs would actively communicate with miRNA through a "ceRNA language" composing vast network of interactions and controlling their expression level [22]. While, short-stranded RNA called microRNA (approximately 22nt) suppresses the expression of mRNA by either inhibiting protein translation or destroying the target mRNA [23].The ceRNA hypothesis holds that ceRNA regulates the expression of transcripts by competing with mRNA for the same microRNA response elements (MREs). Regardless of whether it encodes a protein, RNA transcripts can compete with each other for binding to microRNA and regulate each other, thus forming a huge ceRNA network (ceRNETs) [24].

Several circRNAs have been identified via high-throughput RNA sequencing in various tissues, such as the hypothalamus [25], pituitary [26], uterus [27], and oviduct [28]. Many recent studies have shown that circRNAs may have unique and important functions during embryonic development, and their expression is the most abundant and complex in the cerebral cortex on the 60th day of pregnancy [29]. Study of Zhang et al. determined key hypothalamic circRNAs in sheep and found oar_circ_0012110 can reduce the level of oar-miR-665-3p by acting as miRNA sponge, in turn, regulate the expression of *DIAPH1* gene which function in mediating GnRH pulsatile secretion [25]. Another example of novel_circ_0000417 that competitively bind with miR-136 and promote the apoptotic process in human granulosa cells [30]. In ovine oviduct, novel_circ_0017815 determined as a molecular sponge for oar-miR-181a and regulate the estradiol synthesis and follicular apoptosis [28]. ceRNA study on porcine follicles have highlighted the role of circINHA as a sponge for miR-10a-5p, thus upregulates the expression of connective tissue growth factor (CTGF) and prevent granulosa cells from apoptosis [31]. Chi-circ_ 0,008,219 control the ovine follicles growth and development through competitively bind with miR-468-3p, miR-84c-5p, and miR-483a [32, 33]. CircDDXlO regulates the expression of sirtuin 3 (SIRT3) by inhibiting the activity of miR-1301-3p or miR-4660 and eventually slow down the ovarian aging in humans [34]. CircRNA 11,396 acts as a molecular sponge for miR-187 in bovine cumulus cells to upregulate the expression of BMPR2 (bone morphogenetic protein 2 receptor) antibody [35]. Above discussion suggested that circRNAs have a specific effect on fetal development. Despite substantial study into the prediction, quantification, and annotation of circRNAs based on competing endogenous model, in-depth understanding of the molecular mechanisms of ovarian-related functions are important to study the reproductive traits of Small Tail Han sheep. It is valuable for us to investigate the critical role of ovarian circRNAs as a ceRNA in sheep fecundity. However, sheep-fecundity-associated circRNAs, miRNAs, mRNAs regulatory networks remain unknown.

Small Tail Han (STH) sheep, a well-known Chinese sheep breed, is endemic polytocous with year-round estrus and has an average litter size of 2.29, making it a suitable model to investigate the molecular genetic mechanisms linked with high prolificacy [36, 37]. In comparison, Dolang sheep, which are widely bred in the south Xinjiang region of China [38], have strong adaptability but low fecundity compared to Small Tail Han sheep [39–42], with an average litter size of 1.4 [43, 44]. Multiple studies have been conducted by researchers used RNA sequencing to analyze the gene expression patterns in the ovaries of Small Tail Han sheep during the follicular and luteal phases of the estrous cycle [45–47]. These studies have identified several differentially expressed genes that are involved in follicular development, oocyte maturation, and luteinization, all of which are important for fertility and concluded that Small Tail Han sheep have a unique molecular mechanism for regulating ovarian function, which may contribute to their high fecundity. Given the difference in prolificacy between these two sheep breeds, this study aimed to gain an understanding of the molecular mechanisms related to prolificacy and explore the expression of circRNAs as competitive endogenous RNA (ceRNA) in reproduction through a systematic transcriptome analysis, co-expression networking, functional enrichment, and genomic organization to obtain in-depth insights into the structure–function relationship of reproductive trait-associated circRNAs, miRNAs, and their target mRNAs. Therefore, the current study aims to identify the sheep-fecundity-associated circRNAs, miRNAs, and mRNAs regulatory networks in Small Tail Han and Dolang sheep to explore the regulatory mechanisms of the ovarian circRNAs in sheep fecundity.

## Material and methods

### Ethics statement

The experiment was carried out at the Institute of Animal Sciences, Chinese Academy of Agricultural Sciences, with approval from the Animal Care and Use Committee. The Ministry of Agriculture of the People’s Republic of China authorized the conduct of experiment of this study.

### Ovarian tissue samples

Six healthy experimental animals, including three Small Tail Han sheep (*high fecundity group*) and three Dolang Sheep (*as a control- low fecundity group*), were used in this study. All the animals were approximately 2 years old, weighed around 50 kg, and raised under the same environmental conditions. Ovarian tissues were excided during dissection and the samples were collected for RNA sequencing and expression profiling of coding (mRNA) and non-coding RNA (circular RNA, miRNA) to investigate their roles in the ovary. All collected ovary specimen were immediately placed in liquid nitrogen and stored at − 70 °C for total RNA extraction.

### Construction of mRNA, miRNA and circRNA libraries and sequencing

Total RNA was isolated from the excised ovaries using Trizol (Invitrogen), and RNA integrity was assessed using an Agilent 2100 Bioanalyzer (Agilent Technologies, Santa Clara, USA). For library construction, approximately 10 μg of total RNA per sample were used to deplete rRNA using the TruSeq Stranded Total RNA with Ribo-Zero Gold kit, followed by TRIzol extraction. By using the RNA Library Prep Kit, the rRNA-depleted RNAs were then fragmented and reverse-transcribed to produce cDNA libraries, and these libraries were further sequenced on the Illumina sequencing technology (HiSeqTM 2500), generating 150 bp/125 bp paired-end reads. For the miRNA-seq library, RNA fragments of 18–35 nucleotides were isolated and purified using a 15% polyacrylamide gel electrophoresis, and 5ʹ- and 3ʹ-termini were ligated with proprietary indexed adapters. Reverse transcription and low-cycle PCR were carried out to produce enough products for Illumina sequencing.

### Primary analysis

To assess the quality of raw sequences, we used Fast QC 0.11.5 followed by Trimmomatic-0.36 software, which trimmed out low quality reads/bases in different ways from 3' and 5' ends. For miRNA quality control, cutadapt version. 1.14 software used to remove connector sequence from miRNA fastq files, and Q20 quality control was performed via fastx_toolkit version. 0.0.13 software, retaining sequences with q20 of 80% and above only. Reads containing N bases were filtered out using NGSQCToolkit version. 2.3.3, resulting in high-quality Clean Reads for subsequent analysis.

In order to classified small RNA in the sequencing results, we processed clean reads through several databases and libraries. Firstly, we used Rfam (version. 10.1) to identify non-coding RNAs such as rRNAs, tRNAs, snRNAs, and snoRNAs. Next, we aligned the filtered reads from miRNA-seq libraries using bowtie version. 1.1.1 software and conducted a BLAST search against (http://www.sanger.ac.uk/software/Rfam), GenBank (http://www.ncbi.nlm.nih.gov/genbank/) and Repbase databases. To identify known miRNAs, we aligned the reads against miRBase version. Twenty-twp database (http://www.mirbase.org/). For unannotated small RNAs, we used miRDeep2 package version. 2.0.0.8 with the Perl script ‘quantifer.pl’ [48] to predict novel miRNAs and analyzed the expression of known and novel miRNAs. We identified comparable miRNA star sequences using the miRBase database and the hairpin structure of a pre-miRNA. Finally, we identified DE miRNAs using DESeq2 in R [49], and predicted putative target mRNA using miranda to inquire about the functional role of miRNAs [50].

To analyze mRNA expression, we first aligned clean reads from RNA-seq libraries to the Ovis Aries reference genome using HiSAT2 version. 2.2.1.0. We then used StringTie version. 1.3.3b to assemble the reads and splice them into new transcripts. The sequencing reads were aligned to known mRNA transcripts sequences using an mRNA database available at (ftp.ncbi.nlm.nih.gov/genomes/all/GCF_000298735.2_O), and measured expression levels, which were normalized as FPKM by using express version. 1.5.1. We standardize the counts and identified differentially expressed mRNA using Leverage DESeq [51]. Finally, mRNA with |log2fold changes|≥ 1 and *p*-value < 0.05 were identified as differentially expressed genes (DEGs/DEmRNAs).

To predict circRNAs, we first aligned the sequencing reads of each sample with the reference genome using BWA [52] software and generated a SAM file. We then used CIRI version. 2.0.3 [53] and circ software version. 1.2 [54] to predict novel circular RNAs from the unmapped reads. Only circRNA candidates that were identified were further analyzed for subsequent analysis. The expression levels of circRNA candidates were calculated with back-splice junction reads in RPM algorithm to quantify and normalized the number of junction reads counts and fold change by DESeq2 [55]. Finally, filtered the differentially expressed circRNAs according to the difference multiple and the difference significance test with a foldchange > 2, and a *p*-value < 0.5. Also analyzed the enrichment of differentially expressed circRNAs through the annotation information of circRNA source transcripts. Furthermore, analyzed the functional enrichment analysis (GO and KEGG) of target genes involved in ceRNA network.

### Bioinformatics analysis for ceRNA regulatory networking

To investigate the ceRNA relationships between circRNA, miRNA, and mRNA associated with sheep reproduction, Pearson’s correlation coefficient (r) were calculated between miRNA—mRNA and miRNA—circRNA pairs based on the expression levels of mRNAs, miRNAs, and circRNAs. We selected negatively correlated pairs with a *p* value < 0.05 and Pearson’s r  > 0.8 for further analysis in X_LC-vs-D_LC. Further, used miranda (version.3.3a) software to predict binding sites for circRNA-miRNA and miRNA-mRNA interactions. Subsequently, we built a co-expression network among differentially expressed circRNA-miRNA and miRNA-mRNA pairs using shared pairs from predicted pairs from binding sites and predicted pairs from the expression of mRNA, circRNA, and miRNA through metscape plugin in cytoscape (version. 3.5.1). Similarly, calculated Pearson’s r between differentially expressed circRNA-mRNA pairs and screened out positively correlated pairs based on the role of mRNA-circRNA in the ceRNA relationship. Based on the principle for ceRNA prediction, only used shared pairs of miRNA-mRNA and miRNA-circRNA to predict ceRNA score in conjunction with hypergeometric distribution calculations using the MUTAME [56] method. The smaller the *p*-value corresponding to the ceRNA relationship, the more significant the miRNAs shared between two ceRNAs (mRNA and circRNA). We considered the shared pairs from predicted pairs of circRNA-mRNA based on the expression of circRNA and mRNA Pearson's correlation coefficient, and the predicted pairs of circRNA-mRNA based on ceRNA score principle as the true ceRNAs.

### ceRNA network construction, phylogenetic tree & enrichment analysis of mRNA in ceRNA

CircRNAs and mRNAs communicate through shared miRNAs, which suggests that they may have similar functions. Using the co-expression data obtained in the previous step, we mapped ceRNA regulatory networks via Cytoscape software (version. 3.5.1) for the obtained ceRNA pairs that shared miRNA mutually. The full-length amino acid sequences of target mRNAs involved in ceRNETs were retrieved from NCBI database (https://www.ncbi.nlm.nih.gov/). To align protein sequences, the ClustalW tool (version. 2.0) was used with the default settings, and then manually modified in MEGA version. 7.0. Then, in MEGA 7.0 with default parameter, the neighbor joining (NJ) tree was constructed with bootstrap replicates by using the neighbor joining method [57]. We performed functional enrichment analysis (GO and KEGG) of mRNAs contributing to the ceRNA network using the R package cluster profiler [55], with a *p*-value < 0.05 used as the cut-off value for KEGG pathway enrichment analysis.

### Genomic structure, expression profiling and subcellular localization of target genes

The CDS and genomic sequences of target genes were retrieved from NCBI database (https://www.ncbi.nlm.nih.gov/). The CDS and genomic sequences were used to draw picture of exon/intron organization at Gene Structure Display Server (GSDS) Program (http://gsds.cbi.pku.edu.cn/). To analyze the tissue specific expression profiles of target genes acquired from transcriptome analysis of ovarian tissues of two sheep breeds (X_LC and D_LC), we used FPKM values from RNA-seq data. The heat map along with Phylogenetic tree was generated using TBtools version. 1.108 [58] for differential expression analysis. Subcellular localization prediction of ovarian tissues associated target genes of ceRNA, binding cites was carried out by using several websites, online servers and tools such as TargetP online web server (http://www.cbs.dtu.dk/services/TargetP/), WOLF-PSORTtool (https://wolfpsort.hgc.jp/), ProtComp (http://linux1.softberry.com/berry.phtml) and CELLO v.2.5 (http://cello.life.nctu.edu.tw/).

### Quantitative (qRT) PCR verification

To validate the expression of differentially expressed circRNAs, miRNAs, and mRNAs involved in ceRNA networks of X_LC-vs-D_LC, we used quantitative real-time PCR (qRT-PCR). We randomly selected five circRNAs, miRNAs, and mRNAs for validation. The expression levels of the selected circRNAs, mRNAs, were normalized against the expression of a housekeeping gene, GAPDH and U6 was used as reference gene of miRNA respectively. Primers were designed and synthesized by Sangon Biotech Co., Ltd. (Shanghai, China). Total RNA was extracted using RNA extraction reagent RNAisoPlus (TakaRa, AA6620-1). For circRNAs, Lan Yi bio reverse transcription kit (Lan Yi Biologics, LY-0160) and miRNA first strand cDNA synthesis Kit (tail addition method) (Sheng Gong, B532451) for miRNA were used for reverse transcription. Quantitative real-time polymerase chain reaction (qRT-PCR) was performed in the lightcycler 96 system (Roche Applied Science, Mannheim, America) using SYBR green real-time PCR master mix (Chun Lei Jie Chong, MR2001) for circRNAs and miRNA fluorescent quantitative PCR Kit (Dye Method) (Sango, B532461) for miRNA following the manufacturer’s instructions. The qRT-PCR analysis was carried out in triplicate. The total 20 μL reaction mixture contained 5 μL 2 × iTaq™Universal SYBR@ Green Supermix, 1 μL cDNA, 10μL H2O, and 0.2 μL each of forward and reverse primers. The following programs were used; pre-incubation 95 °C for 3 min, amplification (40 cycles) of 95 °C for 3 s, 60 °C and for 20 s, then melting curves 95 °C for 15 s, 60 °C for 15 s, and 95 °C for 15 s. The relative change of gene expression of circRNAs, miRNAs, and mRNAs among the control and experimental groups was calculated by the 2 − ∆∆Ct method.

### Statistical analysis

All the data presented here mean values ± standard deviations. Student’s T-test was performed for the comparison, and *P*-value < 0.05 was considered as statistically significant.

## Results

### Identification of circRNAs, miRNAs, mRNAs in Ovary Tissue of X_LC-Vs-D_LC

**Fig. 1:**
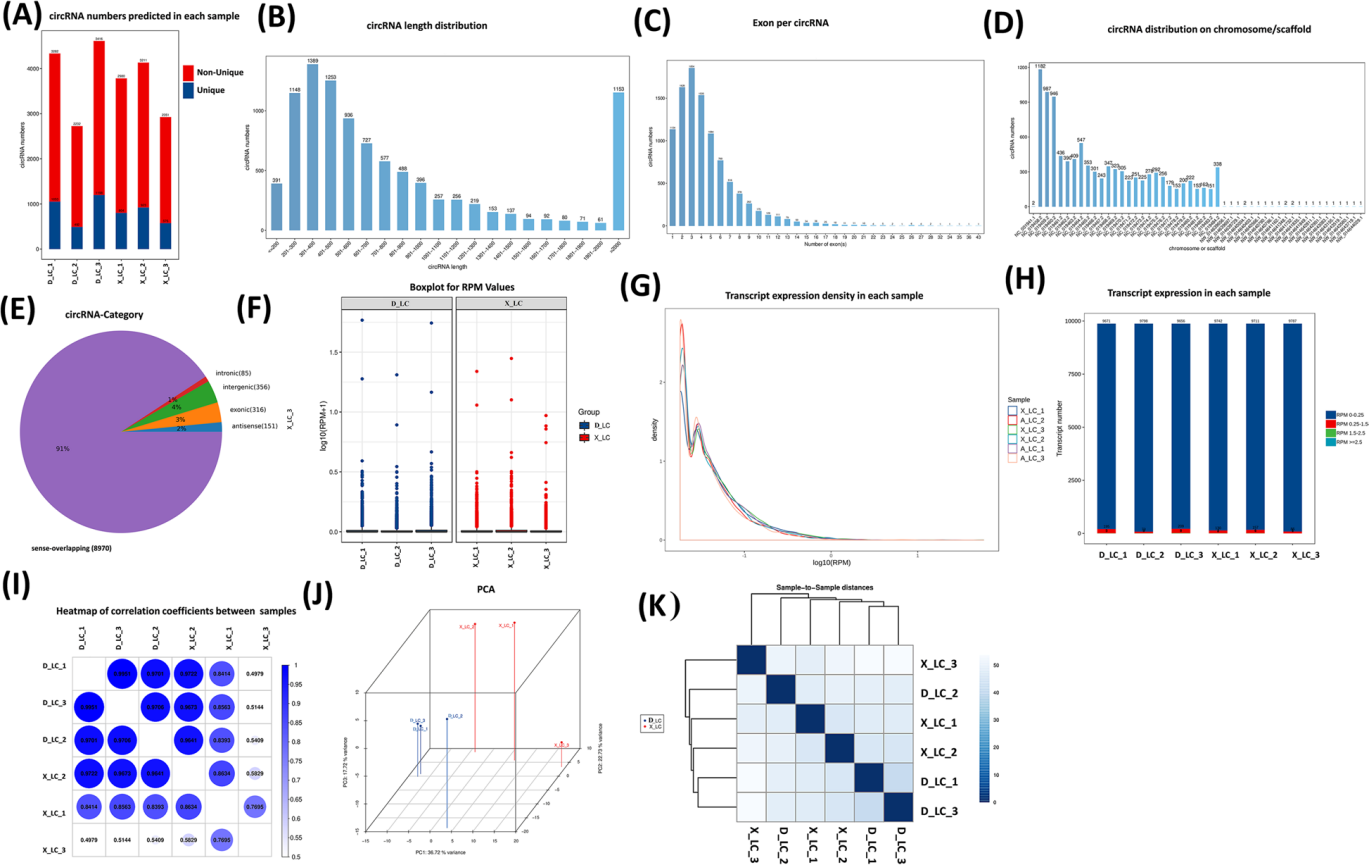
The proportion and characteristics of various circRNAs identified in ovarian tissue of Small Tail Han Sheep X_LC and Dolang Sheep D_LC. **A** Number of CircRNAs Predicted in each sample. Unique circRNAs numbers refers to the number of circRNAs specifically predicted in each sample compared to other samples in the project. **B** Length distribution of CircRNAs sequence. **C** CircRNAs exon number distribution map. The horizontal axis is the number of exons contained in the circRNA, and the longitudinal axis is the number of circRNAs. **D** Plot of the number distribution of circRNAs on individual chromosomes or scaffold. The horizontal axis is the number of chromosomes or scaffold, and the longitudinal axis is the number of circRNAs. **E** Identified circRNAs Origination

We collected ovarian tissue from Small Tail Han sheep (X_LC) and Dolang sheep (D_LC) (*n* = *3each group*) for RNA-seq analysis. We constructed and sequenced 6 ribosomal RNA (rRNA)-depleted libraries, named X_LC (X_LC_1, X_LC_2, X_LC_3) and D_LC (D_LC_1, D_LC_2, D_LC_3). We obtained a total of 91.04 G of clean data, with the effective data volume of each sample distributed between 14.79 and 15.82G. The Q30 base distribution was 91.51 to 92.0%, and the average GC content was 49.23%. We compared the reads of each sample to the reference genome and obtained a comparison rate of 92.44 to 93.29%. Number of circular RNA predicted in each sample. Specifically, in D_LC, we predicted 3,282, 2,232, and 3,416 circRNAs with non-unique numbers and 1,053, 492, and 1,198 with unique numbers in samples D_LC_1, D_LC_2, and D_LC_3, respectively. D_LC_1, D_LC_2, D_LC_3 with non-unique number;3282, 2232, 3416 and unique number; 1053, 492, 1198. In X_LC, we predicted 2,980, 3,211, and 2,351 circRNAs with non-unique numbers and 804, 923, and 575 with unique numbers in samples X_LC_1, X_LC_2, and X_LC_3, respectively (Fig. 1A).

Based on the annotation information of protein-coding genes and transcripts released in the database, the transcript with the largest overlap in position with circRNA obtained through position information comparison, and the sequence of circRNA is predicted based on the transcript information (Table 1). We determined 9878 new circRNAs with a total length of 23,522,667 nt and an average length of 2381.32 nt.

**Table 1:** Statistical table of circular RNA sequence information

Term	All	> = 200nt	> = 500nt	> = 1000nt	N50	Total_Length	Max_Length	Min_Length	Average_Length
circRNA	**9878**	**9493**	**5706**	**2574**	**22,543**	**23,522,667**	**99,455**	**57**	**2381.32**

Among them, majority of circRNAs were characterized with a length of 200–600 nucleotides (nt) and more than 2,000nt (Fig. 1B). The circRNA_4335 predicted with highest circRNA length, whereas circRNA_2452 with the minimum circRNA length. The circRNAs distribution on chromosome identified and obtained top ten chromosomes with maximum number of circular RNAs including; NC_019458.2 (*1182*), NC_019459.2 (*987*), NC_019460.2 (*946*), NC_019461.2 (*436*), NC_019462.2 (*390*), NC_019463.2 (*409*), NC_019464.2 (*547*), NC_019465.2 (*353*), NC_019484.2 (*338*) and NC_019466.2 (*301*) (Fig. 1D). A total of 3,781 parental genes generated these circRNAs, with some parental genes producing single circRNAs and others generating multiple circRNAs. The identified circRNAs were categorized into five types based on their genomic location; including sense-overlapping-8970 (90.81%), intergenic-356 (3.60%), exonic-316 (3.20%), antisense-151 (1.53%), and intronic-85 (0.86%) circRNAs (Fig. 1E). Notably, only a limited number of circRNAs consisted of protein-coding exons. CircRNAs that consisted of one exon were significantly longer than those that comprised multiple exons (Fig. 1B). Identified circRNAs were equipped with various number of exons such as 1854 circRNAs had three exons, 1628 circRNAs had two exons, 1534 circRNAs had four exons, and 1134 circRNAs had one exon (Fig. 1C).

### Differentially expressed circRNAs, miRNAs and mRNAs in X_LC-vs-D_LC ovarian tissue differentiation

**Fig. 2:**
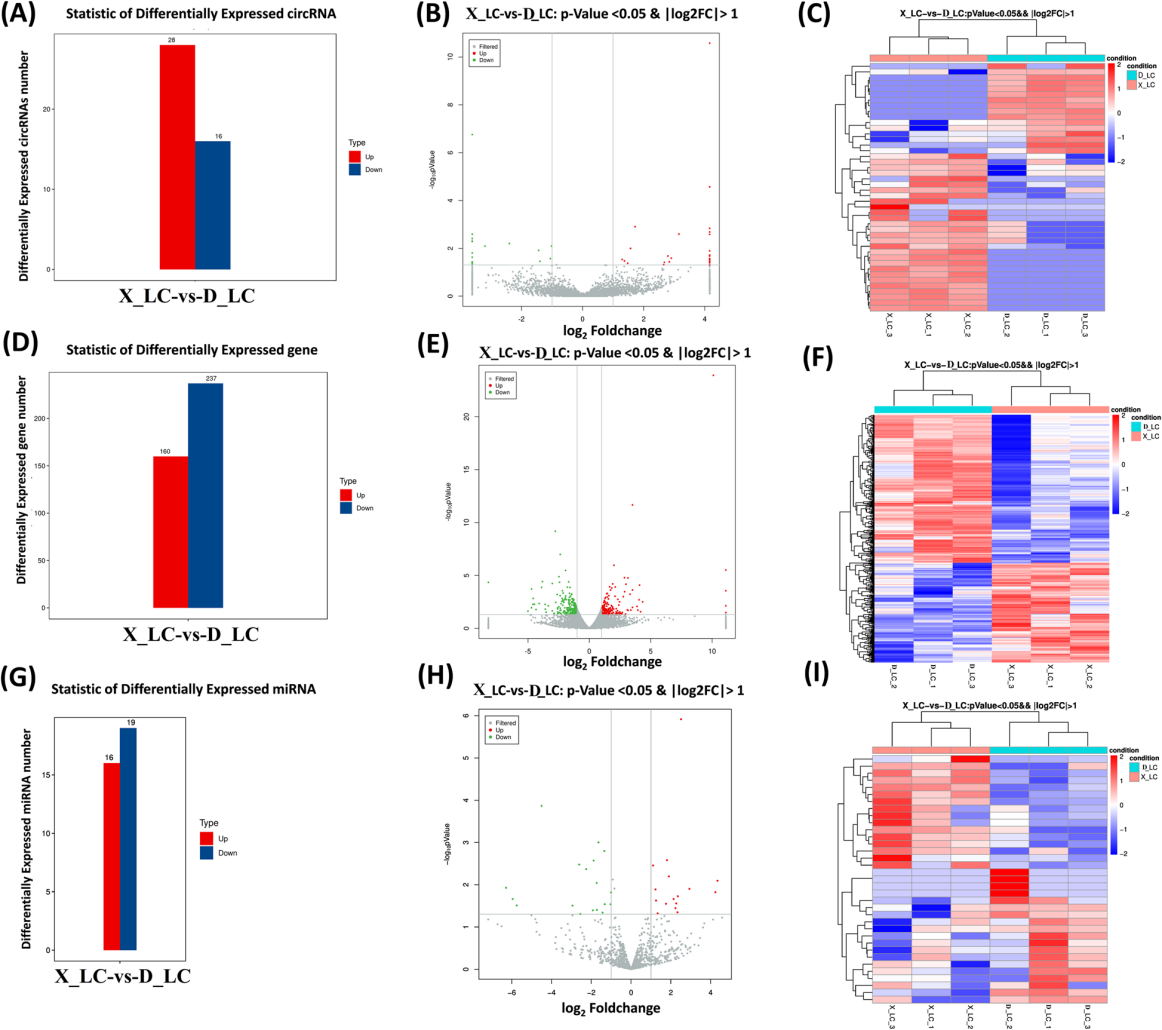
Statistics, volcano representation and expression profiling of noncoding RNAs in X_LC-vs-D_LC. **A-C** Differentially expressed circRNA number, volcano map and heat map representation. **D-F** Differentially expressed mRNA number, volcano map and heat map representation. **G-I** Differentially expressed miRNA, volcano map and heat map representation in Small Tail Han Sheep. The differences resulting from the comparison are reflected in the volcano map, with gray as circRNA with non-significant differences, red and green as the significant differences circRNA, and the horizontal axis as log_2_FoldChange, Y axis direction is -log_10_pValue

By comparison of X_LC-vs-D_LC, a total of 44 DE-circRNAs were identified. The number of significantly upregulated circRNAs was higher than that of significantly downregulated circRNAs in comparison groups (Fig. 2A). For mRNAs, a total of 20,533 mRNAs, including 397 DEGs were screened out with the criteria of differantial-pvauel-0.05 and Foldchange-2. Among them160 DEGs were upregulated and 237 were downregulated respectively (Fig. 2D). In case of miRNAs, 1,186 miRNAs comprising of 146 known miRNAs and 1040 novel miRNAs were identified in X_LC-vs-D_LC. Of these, 35 DEmiRNAs were tested by diff-pval-0.05 and FC-2 in X_LC-vs-D_LC (Fig. 2G). The differences resulting from the comparison were reflected in the volcano map and heat map of differentially expressed circRNA (Fig. 2B-C), mRNA (Fig. 2E-F) and miRNA (Fig. 2H-I) in X_LC-vs-D_LC.

### Co-expression and prediction of target genes of miRNA-mRNA and miRNA-circRNA

**Fig. 3:**
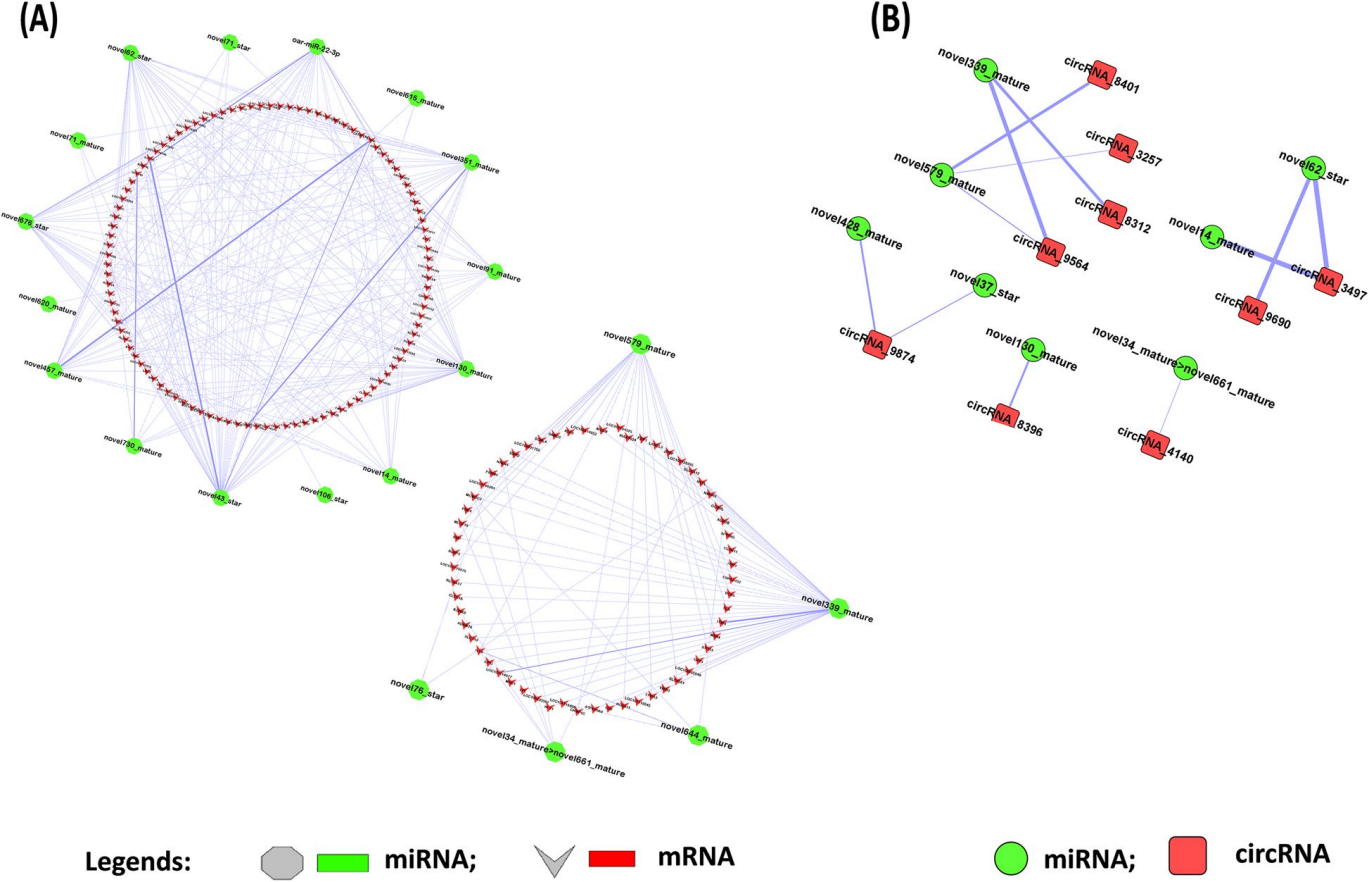
**A-B** Correlation based network between DEmiRNAs and their target DEGs (mRNAs) and DEmiRNAs and their target DECs in ovary tissues

In X_LC-vs-D_LC, correlation (r) was calculated between 35 DEmiRNAs and 397 DE mRNAs (DEGs), resulting in the identification of 2510 miRNA-mRNA correlated pairs with a threshold of an absolute value of r ≥ 0.80 and a *p*-value ≤ 0.05. According to the principle of miRNA and mRNA action, 903 negatively regulated correlated pairs were screened out, and their target binding sites (MREs) on mRNA were further predicted using miranda software version. 3.3a. Thus, we obtained 337 miRNA-mRNA interaction pairs that could be involved in transcriptional degradation/translational inhibition, contributed by 166 DEGs and 22 DEmiRNAs (Supplementary Table 1 and Fig. 3A). Similarly, a correlation coefficient was measured between 35 DEmiRNAs and 44 DECs, resulting in 205 miRNA-circRNA correlated pairs. Following the principle of miRNA and circRNA action, 45 negatively regulated pairs were screened out, and miRNA binding sites (MREs) on circRNAs were predicted using miranda v3.3a (Supplementary Fig. 1). This yielded 12 miRNA-circRNA pairs contributed by 9 DECs and 8 DEmiRNAs (Fig. 3B and Table 2).

**Table 2:** miRNA-circRNA co-expression and prediction of target genes

miRNA	circRNA	R	*p*-value	Total Score	Total Energy	Max Score	Max Energy	Target Length	Positions	MRE
novel579_mature	circRNA_9564	-0.9072255	0.012511391	1885	-152.06	156	-17.28	76,127	31,397 66,000 70,644 43,569 51,686 64,454 35,374 51,517 57,810 64,144 37,643 44,640 65,080	13
novel339_mature	circRNA_9564	-0.9527047	0.003302371	1588	-202.81	164	-25.75	76,127	63,431 38,521 4033 66,980 70,803 15,099 34,764 38,924 12,613 58,194 73,273	11
novel579_mature	circRNA_8401	-0.9430699	0.004769299	1336	-107.02	160	-19.15	25,448	7523 121 4884 9022 10,725 12,785 7594 16,888 4615	9
novel339_mature	circRNA_8312	-0.8283779	0.041653748	1327	-158.59	157	-24.75	33,662	9101 15,594 21,235 14,878 4987 9128 12,990 11,451 19,182	9
novel130_mature	circRNA_8396	-0.8309213	0.040464655	712	-94.96	148	-23.49	44,924	8036 11,410 44,039 16,227 39,995	5
novel62_star	circRNA_9690	-0.8276274	0.042007697	291	-35.27	151	-20.78	1793	527 441	2
novel62_star	circRNA_3497	-0.9782303	0.000705721	140	-20.62	140	-20.62	915	5	1
novel428_mature	circRNA_9874	-0.9387901	0.00550532	144	-23.33	144	-23.33	6583	1691	1
novel579_mature	circRNA_3257	-0.895058	0.015941393	143	-13.78	143	-13.78	758	366	1
novel34_mature > novel661_mature	circRNA_4140	-0.8588374	0.02848384	146	-20.52	146	-20.52	3145	1373	1
novel37_star	circRNA_9874	-0.8587394	0.028522444	145	-15.94	145	-15.94	6583	1797	1
novel14_mature	circRNA_3497	-0.816605	0.04736645	151	-23.54	151	-23.54	915	323	1

### ceRNA data analysis & co-expression analysis and filtering of mRNA and circRNA

**Fig. 4:**
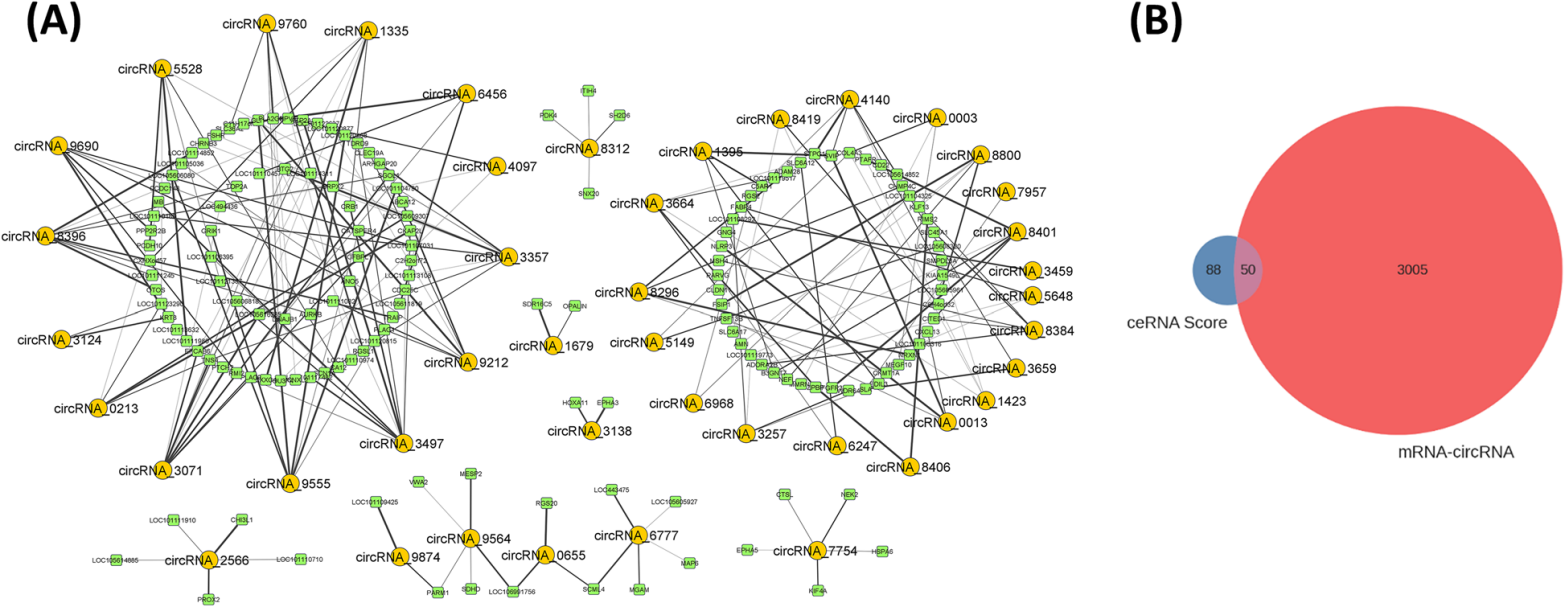
**A** Top 200 Co-expression network of circRNA-mRNA. **B** The results of the co-expression calculation of mRNA and circRNA were used to filter the ceRNA score

Furthermore, the ceRNA score was calculated, resulting in to 88 regulatory relationships between circRNAs and their target genes using the MuTaME method. The smaller the *p*-value corresponding to the ceRNA relationship, the more significant the shared target miRNAs between the two ceRNAs (circRNAs and mRNA). Meanwhile, Pearson correlation was determined among 397 DEmRNAs and 44 DECs in X_LC-vs-D_LC, which led to 4274 pairs of mRNA-circRNA. The co-expression network of the top 200 circRNA-mRNA interactions was constructed (Fig. 4A). Based on the role of mRNA-circRNA in the ceRNA relationship, filtered 3005 positively correlated mRNA-circRNA pairs respectively (Supplementary Table 2). These pairs were combined with the result of ceRNA score, in turn revealing 50 common intersections (Fig. 4B and Supplementary Table 3).

### ceRNA network & phylogenetic tree of ceRNA target genes

**Fig. 5:**
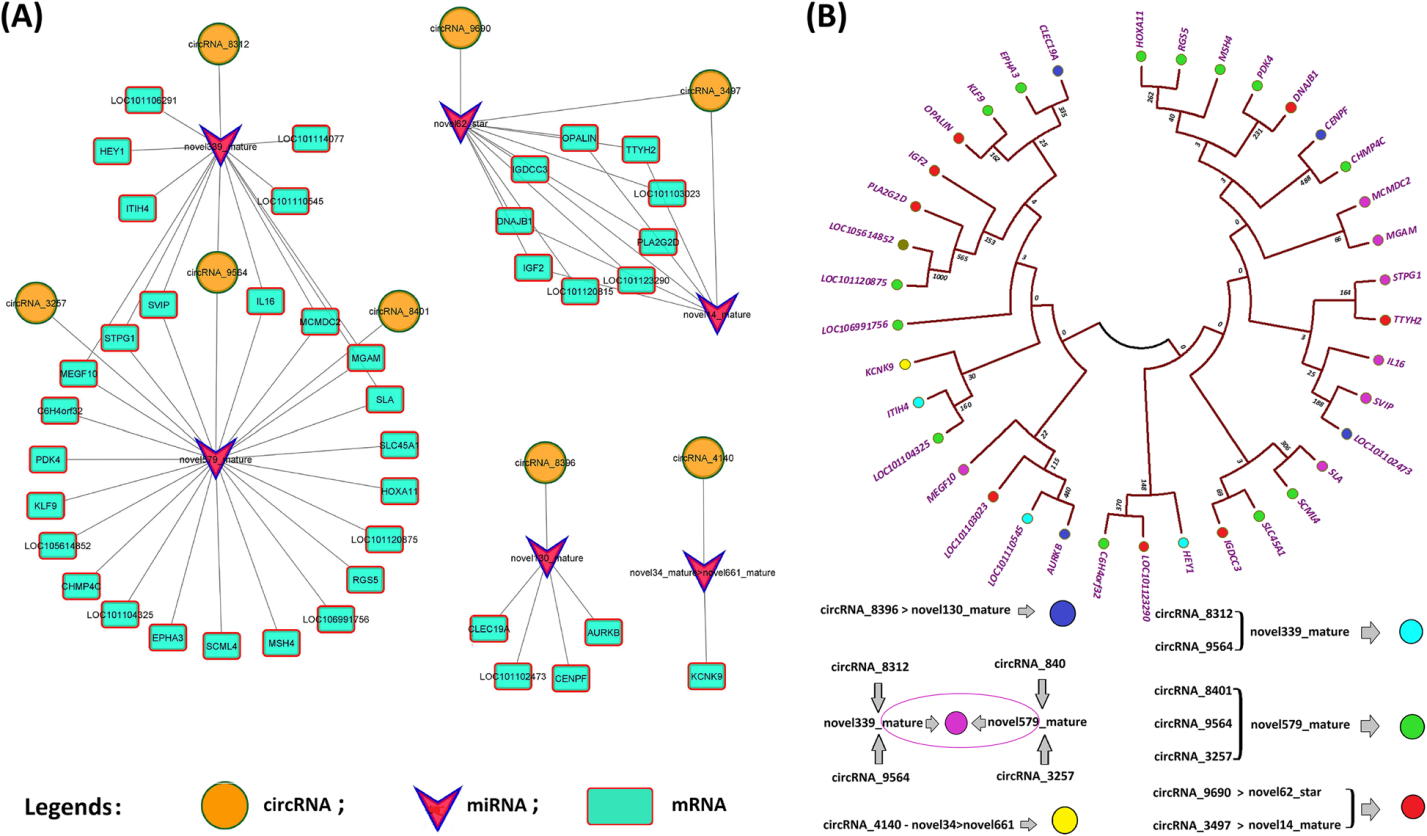
ceRNA network and phylogenetic tree of ceRNA regulatory genes in X_LC-vs-D_LC. **A** circRNA-miRNA-mRNA interaction network in X_LC-vs-D_LC. **B** Phylogenetic tree (Two rooted) constructed with MEGA7 by neighbor joining method. Each node of the tree displays the bootstrap values from 1000 replicates. The tree displayed numerous clades, each clade corresponding to a different evolutionary group, but sharing comparable genes. Different colors in the tree represents ceRNA target genes. novel579_mature was found in circRNAs_8401, circRNAs_9564, circRNAs_3257 and novel339_mature was found in common in circRNAs_8312 and circRNAs_9564 as a common binding site

In accordance with the ceRNA theory, ceRNAs share miRNAs as a junction where upregulated miRNAs are associated with downregulated circRNAs and mRNAs, and downregulated miRNAs are associated with upregulated circRNAs and mRNAs. The ceRNETs were constructed using the obtained top 50 interactions based on ceRNA scores, comprising six miRNAs: novel339_mature, novel579_mature, novel62_star, novel14_mature, novel130_mature, and novel34_mature > novel661_mature; eight circRNAs: circRNA_9564, circRNA_9690, circRNA_3497, circRNA_8312, circRNA_3257, circRNA_8401, circRNA_8396, and circRNA_4140; and 40 mRNAs (Fig. 5A). To understand the evolutionary, structural, and functional associations of ceRNA target mRNAs, a phylogenetic tree was constructed. Clustering into similar clades was observed for genes with similar structures. The best representation was exhibited by novel579_mature, which was common in circRNA_8401, circRNAs_9564, and circRNAs_3257, and novel339_mature, which was common in circRNA_8312 and circRNA_9564 (Fig. 5A and B).

### Functional enrichment analysis of ceRNA

**Fig. 6:**
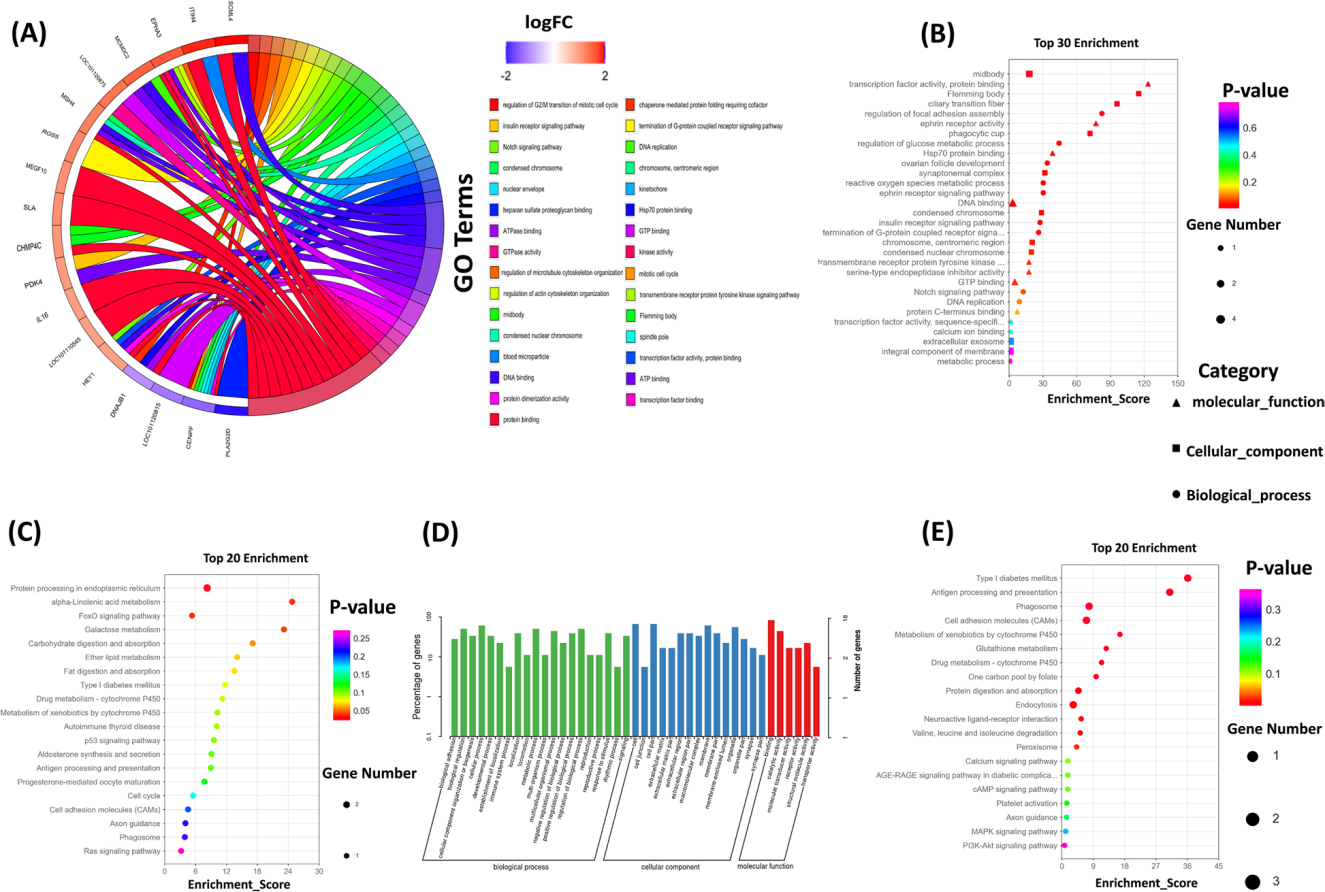
Functional enrichment analysis and of mRNA in ceRNETs and DECs host genes in X_LC-vs-D_LC. **A-B** GO Chord diagram based on logFC and GO bubble chart of ceRNETs regulatory genes. Y axis corresponding to GO entries, X axis corresponding to Enrichment Score, different shapes corresponding to different GO classification (BP, CC, MF). **C** KEGG enrichment analysis of ceRNETs regulatory genes in X_LC-vs-D_LC. X. **D-E** GO and KEGG enrichment analysis of DECs host genes

Gene ontology (GO) analysis of mRNA in ceRNETs revealed that out of 40 mRNAs, 28 were successfully annotated, providing insight into biological processes (BP), cellular components (CC), and molecular functions (MF). Fisher’s exact test was used to calculate the enrichment significance of each term in BP, CC, and MF, with detailed results provided in Supplementary Table 4. Each term is arranged in ascending order according to *p*-value, indicating that a smaller *p*-value corresponds to more significant enrichment. A total of 65 terms had a *p*-value ≤ 0.05, with the first three terms having the smallest *p*-values being "midbody" (TermID: GO:0,030,496, *p*-value: 0.0054) and "transcription factor activity, protein binding" (TermID: GO:0,000,988, *p*-value: 0.0081). Among these, 43 terms had *p*-values ≤ 0.05 in BP, 10 terms in CC, and 12 terms in MF, with none having FDR ≤ 0.05 (Supplementary Table 4 and Fig. 6A-B).

In the KEGG enrichment analysis, only 12 out of 40 mRNAs in ceRNETs were annotated, participating in 40 different pathways. We selected the top 20 pathways with *p*-values ≤ 0.05. Among them, the pathways with the smallest *p*-values were "Protein processing in the endoplasmic reticulum" (TermID: path: oas04141, *p*-value: 0.023) followed by "Alpha-linolenic acid metabolism" (TermID: path: oas00592, *p*-value: 0.030), as shown in Fig. 6C. The majority of pathways were associated with genetic and metabolic pathways, such as galactose metabolism, metabolism of xenobiotics by cytochrome P450, drug metabolism-cytochrome P450, type I diabetes mellitus, antigen processing and presentation, progesterone-mediated oocyte maturation, cell cycle, cell adhesion molecules (CAMs), axon guidance, phagosome, Foxo signaling pathway, p53 signaling pathway, and Ras signaling pathway. Furthermore, we observed similarities between the functional enrichment analysis of DECs host genes and the ceRNA target genes (Fig. 6D-E).

### Gene structure, expression profiling and subcellular localization of target DEGs

**Fig. 7:**
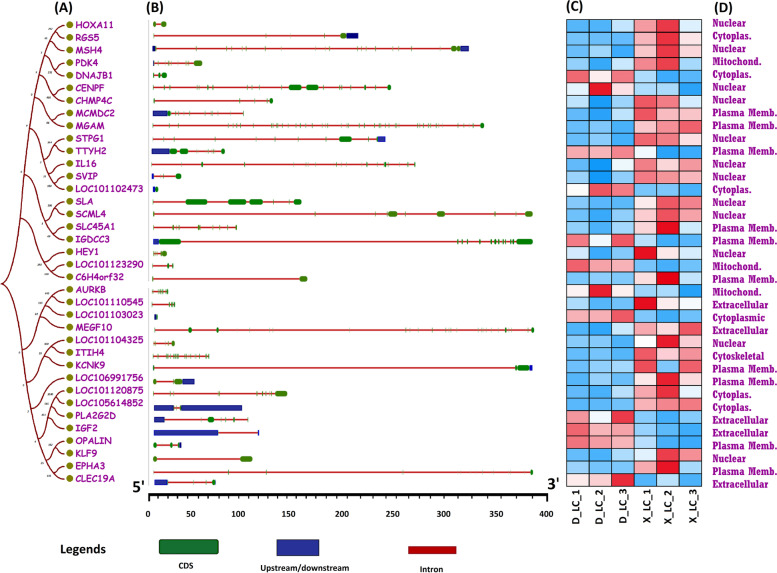
ceRNETs regulatory genes from the ovary tissue of two sheep breeds (**A**) depicts a phylogenetic tree constructed by using the neighbor-joining method and 1000 repeats in MEGA7 software. **B** Different numbers of exons and introns are represented in the gene structure. **C** Expression profiling of fecundity associated genes in two sheep breeds (X_LC) and treatment group (D_LC). Heat map showing the expression of target genes in two group expression profile on the basis of FPKM value. Each gene is colored based on the log_10_ base mean expression. **D** Subcellular localization of ceRNA target genes

Most animal genes contain exons and introns, and their arrangement can reveal their evolutionary relationships and suggest their involvement in specific functions [59]. To further elucidate the evolutionary, structural, and functional relationships of ceRNA target genes, we conducted a comprehensive comparative study by constructing a phylogenetic tree and analyzing gene structures, tissue-specific expression patterns, and predicted subcellular localizations. The compelling illustration in Fig. 7A, B, C, D demonstrates the interconnection between gene structures and subcellular localization. Previous studies have reported a relationship between exon and intron distribution patterns and their biological roles [60, 61]. In this study, we identified SCML4, IGDC33, MEGF10, KCNK9, and EPHA3 as the longest genes compared to LOC101103023, which has the shortest genomic length of 3 Kb. To gain further insight into the structural evolution of the aforementioned genes, we developed a new phylogenetic tree and examined their structural features using the online web portal GSDS (http://gsds.cbi.pku.edu.cn/). Comprehensive analysis of exon–intron structures aligned with the phylogenetic tree sequence provides a holistic illustration of their relative lengths (Fig. 7B) and demonstrates their random distribution throughout the tree. However, clustering into similar clades was observed for genes with similar structures. The best representation was exhibited by novel579_mature, which was common in cirRNA-8401, cirRNA_9564, and cirRNA_3257 (Figs. 5B and 7A). The number of exon regions varies among all the genes (from 1 to 18); however, surprisingly, all closely associated genes exhibited similar structural organization and divergent exon–intron lengths.

To elucidate the potential difference in fecundity between X_LC and D_LC, we compared the mRNA expression profiles of target genes involved in the ceRNA regulatory network (Fig. 7C). A prominent difference was found in gene expression profiles between Dolang sheep (D_LC) and Small Tail Han Sheep (X_LC). EPHA3, KLF9, SLA, SLC45A1, and SCML4, the target genes of novel579_mature, are highly expressed and upregulated in the ovary tissue of Small Tail Han sheep compared to Dolang sheep. Similarly, differential expressions of LOC101102473, PLA2G2D, and LOC101110545 were noted during tissue-specific expression analysis of ovary tissues from both groups. It is evident that our results from tissue-specific expression profiling are in agreement with the ceRNA network, GO analysis, and KEGG enrichment analysis, which confirms the reliability and accuracy of our transcriptome data analysis. These findings are further corroborated by qRT-PCR analysis in the next step.

In order to predict the subcellular localization of ceRNA target genes, we used several websites and online tools to ensure the accuracy of results. Collectively, data suggested that the majority of the aforementioned genes were localized to the cytoplasm, the nuclear envelope, and the plasma membrane. Although few genes were also predicted in mitochondria, cytoskeletal and extracellular membranes. This is consistent with their associated functions (Fig. 7D). It has been observed that most of the circRNA_3257, circRNA_8312, circRNA_8401, and circRNA_9564 based target genes were grouped in same clades and represented similar subcellular localization along with analogues expression profiles. However, novel579_mature was identified as a common miRNA binding site of circRNAs_3257, cirRNA_8401 and circRNAs_9564 and contains the similar target genes. Novel339_mature have a target binding site for circRNAs (circRNAs_8312, circRNAs_9564) circRNAs_8396, circRNAs_9690 and circRNA_4140 have a binding site for novel130_mature, novel62_star and novel34_mature > novel661_mature, indicating the precision and accuracy of our transcriptomic study, revealing strong collaboration of gene structures and their subcellular localization with tissue specific expression patterns along the phylogenetic tree (Figs. 5B and 7A-D).

### Validation of candidate circRNAs

**Fig. 8:**
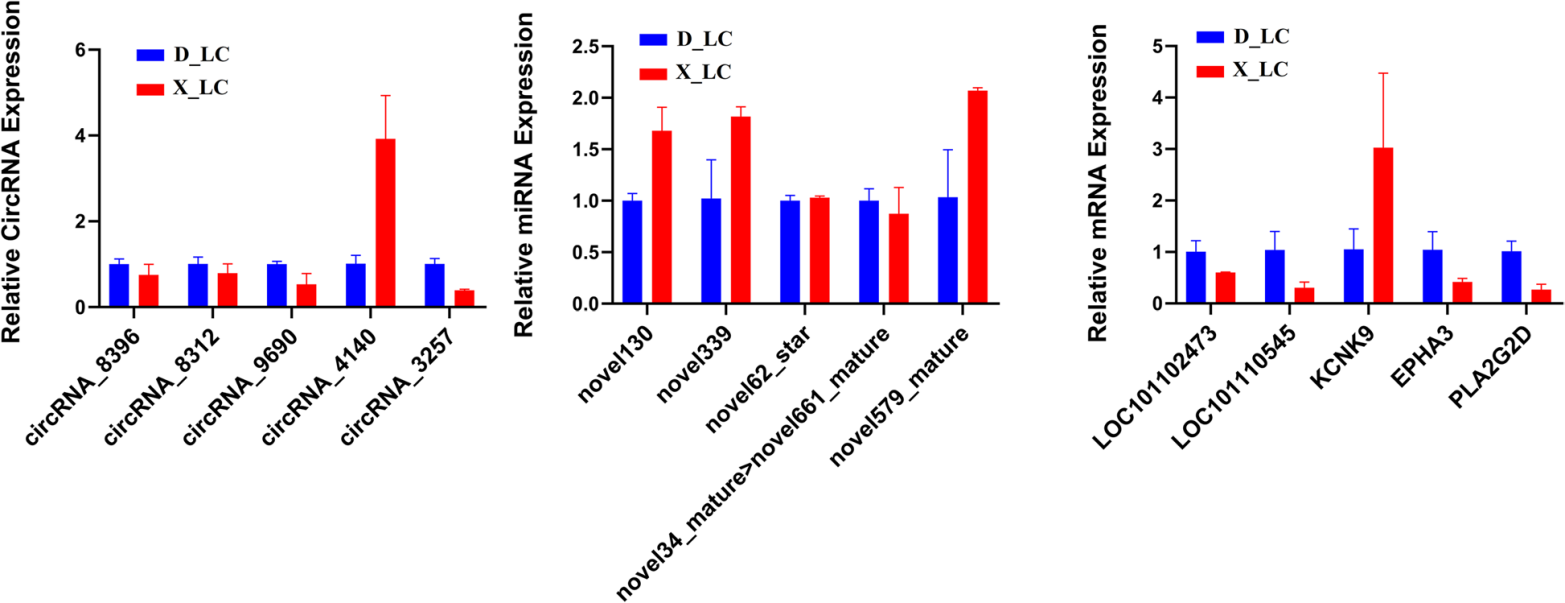
The qRT-PCR verification result of differentially expressed circRNAs, miRNA, mRNA involved in ceRNA network in ovarian tissue of Small Tail Han sheep and Dolang sheep (X_LC-vs-D_LC)

Based on our predicted ceRNA circRNA-miRNA-mRNA interaction network and ceRNA target mRNA KEGG enrichment analysis, we selected key circRNAs (circRNA_8396|NC_019477.2:26838353_26929260_ + , circRNA_8312|NC_019477.2:3206957_3240924_-, circRNA_9690|NC_019484.2:62838118_62839910_-, circRNA_4140|NC_019463.2:66446753_66449897_-), miRNAs (novel130_mature, novel339_mature, novel62_star, novel34_mature > novel661_mature, novel579_mature), and mRNAs (LOC101102473, LOC101110545, KCNK9, EPHA3, PLA2G2D) for expression validation in the ceRNA regulatory network. We performed qRT-PCR and observed dynamic expression profiles of the aforementioned circRNAs, miRNAs, and mRNAs. The results showed downregulation of circRNA_8396 and circRNA_8312 in X_LC-vs-D_LC, whereas circRNA_3257 and circRNA_9690 exhibited significant downregulation, and circRNA_4140 demonstrated remarkable upregulation in ovary tissue of X_LC-vs-D_LC (Fig. 8). In the case of miRNAs, novel130_mature, novel339_mature, and novel579_mature were significantly upregulated, but novel62_star was not significantly upregulated in X_LC ovary tissue. Conversely, novel34_mature > novel661_mature was downregulated in X_LC (Fig. 8). The qRT-PCR results for mRNA revealed that the expression of LOC101102473, LOC101110545, EPHA3, and PLA2G2D were remarkably downregulated in X_LC. However, KCNK9 was significantly upregulated in X_LC ovarian tissue (Fig. 8). These findings were consistent with our predicted results from the transcriptome data.

## Discussion

### Differentially expressed circular RNAs and their functional enrichment analysis 

A large body of knowledge has emphasized the significant role of noncoding RNAs in sheep fertility [32, 62–64]. The study of ceRNA consider landmark in understanding the mutual regulatory relationship and interactions of RNA-RNA [64, 65]. In this study, high-throughput sequencing was used to identify and characterize ovarian tissue circRNAs from Small Tail Han Sheep (X_LC) and Dolang sheep (D_LC) and determined ceRNA regulatory network. In this study, majority of circular RNAs were predicted with exon 1–5 which are consistent with the sheep uterus circRNAs [27] but inconsistent with sheep pituitary gland circRNAs [26] thereby exhibit complexity and functional diversity. Besides, studies have been reported about the expression and potential biological functions of circRNAs in reproductive organs in goats [66], mice [67] and humans [68]. Several findings have been reported about the circRNAs expression and their biological functions as miRNA sponge [25, 27, 28]. In sheep uterus study, 147 and 364 circRNAs out of 32,687 were differentially expressed in polytocous and monotocous groups in the follicular phase and luteal phases and DECs host genes significantly enriched with estrogen signaling pathway, oxytocin signaling pathway [27]. While, in the comparative study between the follicular phase/luteal phase of sheep, 15 DECs out of 3223 were predicted and DECs host genes were enriched in the Rap1 signaling pathway, PI3K–Akt signaling pathway and neuroactive ligand–receptor interactions [28]. In this study, we found 9,878 new circRNAs with a total length of 23522667nt and an average length of 2381.32nt respectively. 44 circRNAs were differentially expressed and majority of enriched GO terms of DECs host genes were related to cell proliferation and reproductive process and KEGG enrichment analysis enriched in cell adhesion molecules, phagosome, PI3K-Akt signaling pathway, neuroactive ligand-receptor interaction, glutathione metabolism, metabolism of xenobiotics by cytochrome P450, MAPK, axon guidance, valine, leucine and isoleucine degradation, and endocytosis pathways. A similar phenomenon was observed in other studies [27, 28, 32]. Metabolic changes reported during transition period from ovulation to the estrous and biomolecules including vitamins, amino acids, lipid, benzoic acid, carbohydrates and other intermediate and secondary metabolites are at their highest levels at the time of ovulation [27, 69, 70]. Prior stud have been reported that MAPK pathway have a great effect on granulosa and cumulus cells which plays essential role in oocyte maturation [71]. Therefore, based on above evidences we suggested that DECs might contribute in sheep prolificacy.

### CeRNA analysis and networking/ functional enrichment analysis of ceRNA regulatory genes 

Circular RNAs (circRNAs) as ceRNA contribute to a various signaling pathways that are crucial for the development process [72]. The fact that mRNA expression negatively regulated by miRNA activity, whereas, circRNAs inhibit or relieve repression of miRNA for translation [73]. Study determined the role of circRNA as ceRNA in follicular development in Hanper sheep [32]. Here, circRNA and mRNA target binding sites were predicted for miRNA (Fig. 3A-B and Table 2) which benefited to determined regulatory relationship among differentially expressed circRNAs, miRNA, mRNA (ceRNETs) (Fig. 5A). In this study, numerous circRNAs holding binding site for common miRNA such as; circRNA_9564, circRNA_8312 shared novel339_mature miRNA, circRNA_9564, circRNA_8401, circRNA_3257 shared novel579_mature miRNA respectively. Whereas, circRNA_8396, circRNA_9690, circRNA_4140 have potential binding sites for only single miRNA including novel339_mature, novel62_star, and novel34_mature > novel661_mature respectively. novel579_mature dominantly target to the number of mRNAs including; CHMP4C, EPHA3, SCML4, MSH4, LOC106991756, LOC101120875, HOXA11, RGS5, SLC45A1, C6H4orf32, LOC101104325, and PDK4 which significantly involved in pathways including endocytosis and axon guidance pathway. Prior study determined the crucial role of endocytic pathways in the development of the reproductive organs [74]. circRNA_8396 have potential binding sites for novel339_mature which target to the following mRNAs; CENPF, CLEC19A, AURKB, and LOC101102473.circRNAs_4140 have potential binding sites for novel34_mature > novel661_mature which target to KCNK9. To further investigate potential circRNAs mechanisms of action in ceRNA regulatory network, we applied KEGG analyses. It was reported that foxO signaling pathway, cell cycle, p53 signaling pathway, endocytosis, progesterone-mediated oocyte maturation participate in reproduction [75]. The results we obtained suggest that multiple signaling pathways form a complex regulatory network involved in prolificacy.

### Coherence of ceRNA regulatory network with differential expression datasets to regulate reproduction

CircRNAs biological function as a microRNA (miRNA) sponge and regulating the target mRNA expression by forming the circRNA-miRNA-mRNA regulatory axis [60]. Pearson correlation based networking and expression profiling provided ceRNA regulatory pairs as shown in figure (Fig. 5A). KEGG enrichment analysis of mRNA involved in ceRNETs revealed significant pathways such as; Galactose metabolism, Glutathione metabolism, Metabolism of xenobiotics by cytochrome P450, Drug metabolism—cytochrome P450, Cell adhesion molecules (CAMs), Antigen processing and presentation, p53 signaling pathway, and Progesterone-mediated oocyte maturation pathways respectively. Of note, studies have demonstrated the involvement of above-mentioned pathways in ovarian physiology and play essential role in follicular development, oocyte maturation, development of reproductive organ, proliferation, immunity, antioxidant, and metabolic process [74–77]. Previous studies demonstrated that the exposure of xenobiotics may destroy the primordial follicles which is responsible for premature ovarian failure and reduce fertility [78, 79]. Glutathione metabolism, well known for their spectacular role as a free-radical scavenger, intervenient in xenobiotics metabolism, cell-cycle regulation, and a reservoir of cysteine [80], as well as play key roles in cellular redox homeostasis [81]. Study reported the involvement of Progesterone-mediated oocyte maturation pathways in oocyte development in sheep breeds [82],and regulating the uterine receptivity and maintenance of pregnancy [83].

Researchers established the role of galactose metabolism in energy delivery, and galactosylation of complex molecules [84]. The metabolism of galactose to UDP-glucose involves three major enzymes, galactokinase, galactose-1-phosphate uridyltransferase (GALT), and UDP-galactose-4-epimerase. In case of any disturbance or deficiency of UDP-galactose are considered to be important in the pathogenesis of ovarian dysfunction and in GALT deficiency [85] due to interference with ovarian apoptosis and gonadotrophin signaling, thus effecting fertility. Wu et al. reported that deregulation of glucose metabolism in diabetic individuals [86], which might induce ovarian anomalies [87]. It is believed that these pathways may play a critical role in regulating ovarian physiology. Previously, study have also examined miRNAs as important components of the p53 transcriptional network [88]. MiR-25 and miR-30d, have been shown to negatively regulate the transcription of P53 gene. Several other miRNAs, including miR-16–1, miR-143, miR-145, miR-34, miR-194, miR-192, miR-215, and miR-29, have been identified as miRNAs that are involved in the transcription P53 network, either by being directly altered by p53 or through their associations with downstream genes targeted by p53[89].

### Significance of ceRNA target gene in regulating reproduction and associated metabolic syndrome

Through ceRNA circRNA-miRNA-mRNA interaction analysis and based on functional enrichment analysis established the following key ceRNA regulatory axis; circRNA_3257-novel579_mature-EPHA3, circRNA_8396-novel130_mature-LOC101102473, and circRNA_4140- novel34_mature > novel661_mature-KCNK9. These shortlisted circRNAs were the result of sense-overlapping and the length of circRNA_3257, circRNA_4140, circRNA_8312, circRNA_8396, circRNA_9690, circRNA_9564 were 758, 3145, 33,662, 44,924, 1793, and 76,127 nt respectively. We found that downregulated circRNA_3257 had a potential binding site for upregulated novel579_mature miRNA and influences EPHA3 gene expression, which is regulated by the protein kinase A (PKA) pathway [90]. The ephrin-Eph gene family has a known physiological role in regulating mammalian reproductive function, such as in granulosa cells of bovine ovarian follicles [91], mouse [90], and human luteinizing granulosa cells [92]. Prior studies have suggested that EPHA3 plays a role in treating ovarian endometriosis, potentially promoting apoptosis and autophagy of macrophages via the inhibition of the mTOR signaling pathway and reducing oxidative stress [93, 94]. In this study, we found that downregulated EPHA3, involved in the axon guidance pathway, functions through cell-to-cell contact [95], and regulates the expression of guidance proteins Ras and Rho GTPases during embryonic development [96]. Furthermore, EPHA3 has been shown to interact with presenilin genes sel-12 (PS1), regulating axon guidance and kinesin-mediated axonal transport of motor neurons. Loss of function mutations in the presenilin genes sel-12 results in abnormal axonal projections, an effect attributed to altered Notch signaling pathway [97], which is integral to maintaining fertility in the ovaries through developmental regulation and granulosa cell function [98]. Based on the ceRNA hypothesis, we suggest that circRNA_3257 may act as a sponge for novel579_mature miRNA, thus favoring the expression of repressed EPHA3, and potentially play a role in the fecundity of Small Tail Han Sheep.

LOC101102473 is a target gene of novel130_mature that is downregulated in ovary tissue of Small Tail Han Sheep [32], and is involved in pathways related to reproduction, such as FoxO signaling pathway, Cell cycle, p53 signaling pathway, Progesterone-mediated oocyte maturation, consistent with the previous research [77]. FOXO signaling is the central pathway controlling growth and metabolism in all cells [99], and closely related with ovarian function [100]. Low levels of p53 expression maintain cell cycle homeostasis and cell death [101]. Dysregulation of the cell cycle, particularly the G1-S-phase transition, is implicated in epithelial ovarian cancer [102]. If DNA damage occurs, p53 accumulates in the cells and induces p21-mediated inhibition of cyclinD/CDK. The transition to S phase is triggered by the activation of the cyclinD/CDK complex, which phosphorylates the retinoblastoma protein pRb, a known cell proliferation regulator [102]. The transcription factor p53 functions as a suppressor of cell growth, and alterations in p53 lead to loss of this negative growth regulation and more rapid cell proliferation. Previous studies have demonstrated that dysfunction of the p53 signaling pathway contributes to the development of ovarian cancers [103], hence affecting fertility. These outcomes suggest that circRNA_8396 might affect sheep fecundity by regulating the expression of LOC101102473 genes linked with the above signaling pathways via sponging novel130_mature miRNA.

The gene KCNK9, which is linked to potassium channels in X_LC-vs-D_LC and has been previously identified in the membrane of cow oocytes [104], is upregulated as a target gene of novel34_mature > novel661_mature. While previous studies have shown its potential role as a therapeutic target for adenomyosis which induce infertility [105]. KCNK9 also helps to control progesterone production in the ovary, and intracellular potassium and calcium concentrations have a significant impact on fertility [106]. Our functional enrichment analysis has revealed that KCNK9 is involved in the aldosterone synthesis and secretion pathway, which is critical for maintaining blood pressure, circulating blood volume, and uteroplacental perfusion during pregnancy [107]. Several other studies have shown that aldosterone synthesis and secretion levels are high throughout pregnancy, indicating potential involvement in the regulation of placental and fetal development [108, 109]. However, studies have also reported that aldosterone is involved in gynecological diseases due to metabolic alterations induced by the usage of hormonal contraceptives, PCOS, uterine fibroids and endometriosis, inflammation, and hypertension, which can result in an increase in the synthesis of angiotensinogen, activating all the RAAS and inducing the onset of sodium and water retention [110]. Therefore, we hypothesize that circRNA_4140 may act as a sponge for novel34_mature > novel661_mature and regulate the expression of KCNK9, which plays a crucial role in fertility.

In addition to the upregulated novel339_mature, the downregulated LOC101110545 (HLA class II histocompatibility antigen, DO alpha chain) gene is also involved in reproduction, as it is a target gene involved in the Cell adhesion molecules (CAMs) [111] and Phagosome [112, 113] pathways crucial for reproduction. CAMs are a family of glycoproteins that play a crucial role in various physiological processes, including cell migration, tissue development, and immune response. They also play key roles in inducing leukocyte infiltration in an inflammatory site during ovulation [114]. Studies have shown that CAMs are implicated in the regulation of ovarian follicular development, oocyte maturation, and fertilization in sheep [115, 116], all of which are critical for successful reproduction and fecundity. One of the key CAMs involved in sheep reproduction is integrin, which facilitates cell–cell and cell–matrix interactions, and is regulated during folliculogenesis and ovulation. Integrin-mediated signaling is essential for the survival and growth of ovarian follicles, as well as for the adhesion and migration of oocytes and granulosa cells during follicle development [115]. Another important CAM in sheep fecundity is selectin. Selectin is another important CAM in sheep fecundity. It is a family of carbohydrate-binding proteins that mediate leukocyte-endothelial cell adhesion during inflammation and immune responses. Studies have reported that selectins play a role in follicular development and ovulation by facilitating the adhesion and migration of leukocytes to the ovary and promoting the release of inflammatory cytokines, chemokines and growth factors [117, 118]. Changes in these molecules are associated with the cyclic changes in the estrous cycle, compounding their role in the ovulatory process [119]. Further research is needed to elucidate the precise mechanisms underlying CAM-mediated signaling in sheep reproduction and to develop novel interventions to improve sheep breeding and production.

In livestock animals, including sheep, phagosomes play a crucial role in regulating reproductive processes such as ovulation, fertilization, and embryo development [120]. As ovarian follicles develop, some follicles undergo atresia, a process by which they degenerate and are eliminated from the ovary [121, 122]. This process involves the death of granulosa cells, which are engulfed and cleared by phagocytes via phagocytosis. Moreover, in mammals, the engulfment of apoptotic cells by macrophages induces the production of anti-inflammatory cytokines to suppress inflammatory responses [123]. Failure to properly clear apoptotic granulosa cells can lead to inflammation and oxidative stress, which can negatively impact follicular development and ovulation [122, 124]. Based on the evidence presented, we can infer that circRNA_8312 may influence LOC101110545 gene expression by suppressing novel339_mature activity. Although the role of the LOC101110545 gene in reproduction has yet to be investigated, our data suggest its possible involvement in reproductive performance through ceRNA networks that regulate ovarian physiology. Comparison between our results and those of previous studies suggests that these selected circRNAs may act as ceRNAs and contribute to signaling pathways regulating reproductive traits. However, further research is required to identify the exact mechanisms involved. This study provides an important source of information that various circular RNAs can act as competitive endogenous RNA, containing common miRNA binding sites, and may act as miRNA sponges to regulate the expression of valuable prolificacy genes, thereby improving sheep fecundity.

## Conclusion

In this study, analyzed the expression of circRNAs, miRNAs, and mRNAs in the ovine tissue of Small Tail Han sheep and Dolang sheep via using RNA sequencing. A total of 44 differentially expressed circRNAs, 397 differentially expressed mRNAs, and 35 differentially expressed miRNAs were identified. We went through a comprehensive study on ceRNA regulatory network analysis of ovine tissues from two different sheep breeds with contrasting fecundity traits. All of the DE circRNAs and mRNAs (fold change > 1, *p* < 0.05), combined with all of the miRNAs (differently or non-DE). By calculating the Pearson correlation between circRNA-miRNA and miRNA-mRNA, we were able to retain the regulatory relationship of circRNA (upregulation)-miRNAs (downregulation or invariably)-mRNA (upregulation) or circRNA (downregulation)-miRNAs (upregulation or invariably)-mRNA (downregulation), and eventually calculated the ceRNA score to identify circRNA–miRNA–mRNA pairs. Based on the role of mRNA-circRNA in the ceRNA relationship, we found a co-expression positive relationship between the differentially expressed circRNA and mRNA, with a correlation coefficient greater than 0.8. A total of 50 significant ternary relationship (circRNAs-miRNA-mRNA) were identified involved in fertility-related pathways such as FoxO signaling pathway, Cell cycle, p53 signaling pathway, Progesterone-mediated oocyte maturation, Cell adhesion molecules, Phagosome, Antigen processing and presentation, and Axon guidance. We identified several key circRNAs, including circRNA_3257, circRNA_8396, circRNA_4140, and circRNA_8312, that act as molecular sponges for miRNAs, and target genes including EPHA3, LOC101102473, KCNK9, and LOC101110545. Our study provides new insights into the molecular mechanisms underlying fertility in sheep and highlights the importance of ceRNA network analysis for understanding complex regulatory networks.

## Supplementary Information

**Additional file 1.**

**Additional file 2: Supplementary Table 1.** Correlation and Target Found between miRNA and mRNA.

**Additional file 3: Supplementary Table 2.** Correlation of circRNA-mRNA.

**Additional file 4: Supplementary Table 3.** ceRNA score of ceRNA regulatory pairs.

**Additional file 5: Supplementary Table 4.** Gene ontology of mRNA in ceRNA regulatory network.

**Additional file 6: Supplementary Table 5.** Primer Sequence of circRNAs, miRNAs, and mRNAs for qRT-PCR.

## Data Availability

The materials and datasets used and analyzed during the present study are available from the corresponding author upon reasonable request.
