# Supplementary figures and images for: Genome wide identification and characterization of fertility associated novel CircRNAs as ceRNA reveal their regulatory roles in sheep fecundity
Source: J Ovarian Res. 2023 Jun 20;16:115. doi: 10.1186/s13048-023-01178-2 (PMC10280924; doi:10.1186/s13048-023-01178-2)

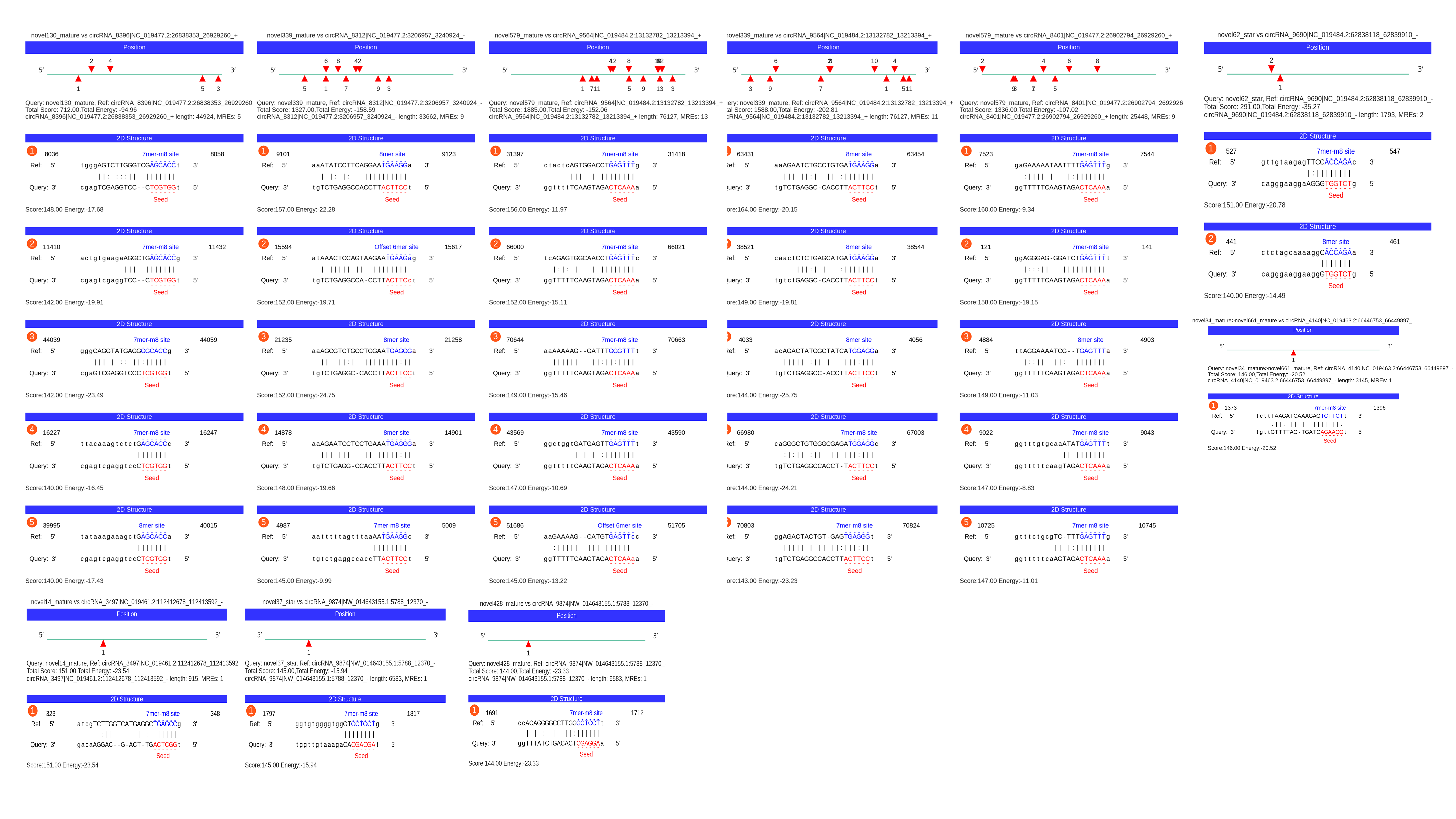

Supplement: Supplementary file 1 — Additional file 1. [file 13048_2023_1178_MOESM1_ESM.tif]
